# Poxvirus MVA Expressing SARS-CoV-2 S Protein Induces Robust Immunity and Protects Rhesus Macaques From SARS-CoV-2

**DOI:** 10.3389/fimmu.2022.845887

**Published:** 2022-03-16

**Authors:** Petra Mooij, Juan García-Arriaza, Patricia Pérez, Adrian Lázaro-Frías, Babs E. Verstrepen, Kinga P. Böszörményi, Daniella Mortier, Zahra Fagrouch, Gwendoline Kiemenyi-Kayere, Henk Niphuis, Roja Fidel Acar, Lisette Meijer, Marieke A. Stammes, Ivanela Kondova, Ernst J. Verschoor, Corine H. GeurtsvanKessel, Erwin de Bruin, Reina S. Sikkema, Joanna Luczkowiak, Rafael Delgado, Dolores Montenegro, Eugenia Puentes, Esteban Rodríguez, Willy M. J. M. Bogers, Gerrit Koopman, Mariano Esteban

**Affiliations:** ^1^ Department of Virology, Biomedical Primate Research Centre (BPRC), Rijswijk, Netherlands; ^2^ Department of Molecular and Cellular Biology, Centro Nacional de Biotecnología (CNB), Consejo Superior de Investigaciones Científicas (CSIC), Madrid, Spain; ^3^ Centro de Investigación Biomédica en Red de Enfermedades Infecciosas (CIBERINFEC), Madrid, Spain; ^4^ Department of Parasitology, Biomedical Primate Research Centre (BPRC), Rijswijk, Netherlands; ^5^ Animal Science Department, Biomedical Primate Research Centre (BPRC), Rijswijk, Netherlands; ^6^ Department of Viroscience, Erasmus Medical Center (MC), Rotterdam, Netherlands; ^7^ Instituto de Investigación Hospital Universitario 12 de Octubre (imas12), Madrid, Spain; ^8^ Department of Medicine, Universidad Complutense School of Medicine, Madrid, Spain; ^9^ Biofabri, O Porriño, Spain

**Keywords:** SARS-CoV-2, COVID-19, spike, MVA vaccine, rhesus macaques, safety, immunogenicity, efficacy

## Abstract

Novel safe, immunogenic, and effective vaccines are needed to control the COVID-19 pandemic, caused by SARS-CoV-2. Here, we describe the safety, robust immunogenicity, and potent efficacy elicited in rhesus macaques by a modified vaccinia virus Ankara (MVA) vector expressing a full-length SARS-CoV-2 spike (S) protein (MVA-S). MVA-S vaccination was well tolerated and induced S and receptor-binding domain (RBD)-binding IgG antibodies and neutralizing antibodies against SARS-CoV-2 and several variants of concern. S-specific IFNγ, but not IL-4, -producing cells were also elicited. After SARS-CoV-2 challenge, vaccinated animals showed a significant strong reduction of virus loads in bronchoalveolar lavages (BAL) and decreased levels in throat and nasal mucosa. Remarkably, MVA-S also protected macaques from fever and infection-induced cytokine storm. Computed tomography and histological examination of the lungs showed reduced lung pathology in MVA-S-vaccinated animals. These findings favor the use of MVA-S as a potential vaccine for SARS-CoV-2 in clinical trials.

## Introduction

Severe acute respiratory syndrome coronavirus 2 (SARS-CoV-2), which emerged in 2019 in Wuhan, China, has rapidly spread across the globe, infecting more than 262 million people, and over 5.2 million people have died due to the coronavirus disease 2019 (COVID-19). Several vaccines have now been approved for human use, including mRNA vaccines (BioNTech/Pfizer, Moderna), adenovirus-vector-based vaccines (AstraZeneca, Johnson & Johnson, CanSino Biologics, Gamaleya), and inactivated virus vaccines (Sinovac Biotech and Sinopharm). These vaccines have shown a good safety profile, potent immunogenicity, high efficacy, and relative durability of immune responses (1). The current vaccines against COVID-19 either express the full-length S protein (AstraZeneca, CanSino Biologics, Gamaleya), or a stabilized prefusion form of the S protein with proline-stabilizing mutations and/or mutation of the furin cleavage site (Pfizer/BioNTech, Moderna, Johnson & Johnson) (1–6). The emergence of variants of concern (VOC), particularly beta and delta variants that showed more resistance to current vaccines than other variants (6–11), together with the inability to fully protect against reinfection and transmission in vaccinees (12), highlights the need for novel optimized SARS-CoV-2 vaccines. In addition, the reported adverse cases of thrombosis with the adenovirus vaccines (13) and myocarditis with the mRNA vaccines particularly in young individuals (14) indicate that a deeper understanding of the mechanisms of action of the vaccines should be undertaken.

We and others have developed several COVID-19 vaccine candidates based on the poxvirus-modified vaccinia virus Ankara (MVA) vector expressing the S protein that have shown a potent immunogenicity profile and full efficacy in several animal models, such as mice (15–21), hamsters (22), and non-human primates (NHPs) (20). MVA is a highly attenuated strain of vaccinia virus, with a well-established safety, immunogenicity, and protective profile in preclinical and clinical research as a vaccine candidate against several infectious diseases and cancer (23–25). MVA-based vaccines are safe and well tolerated, induce potent and durable antibody and CD4^+^ and CD8^+^ T-cell responses, even after a single immunization, are stable, and can be produced at high titer (26–28). Furthermore, MVA-based vaccines can be used for boosting DNA, mRNA, and adenovirus or other viral vector-based vaccines (26, 29–31).

In the present study, we evaluated the safety, immunogenicity, and efficacy in rhesus macaques of a novel MVA-based vaccine candidate expressing a human codon-optimized full-length SARS-CoV-2 S protein (MVA-S), which was previously reported to induce potent B- and T-cell responses and full efficacy in mice (16, 17, 19). The MVA-S vaccine was well tolerated, and strong binding IgG antibody responses, neutralizing antibodies against parental SARS-CoV-2 and VOC and S-specific IFNγ-producing cells, were induced. Upon SARS-CoV-2 challenge, the MVA-S-immunized animals were protected against virus replication in the lungs, had reduced the levels of pro-inflammatory cytokines in serum and BAL samples, and had less lung pathology, compared to control MVA-wild-type (WT)-inoculated macaques. These results reinforce the use of MVA-S as a potential vaccine in clinical trials either alone or in combination with other vaccines.

## Materials and Methods

### Animals and Ethics Statement

The study was performed in twelve adult female rhesus macaques (*Macaca mulatta*) that were divided into two groups of six animals each (MVA-S and MVA-WT groups). The animals underwent a full physical examination prior to entering the study. All individuals were healthy with normal clinical chemistry and hematology levels and free of pathogens. They were negative for antibodies to simian T-cell leukemia virus and simian retrovirus, and negative for binding antibodies to the RBD of the S protein of SARS-CoV2. The study was reviewed and approved by the Dutch “Centrale Commissie Dierproeven” (AVD5020020209404-2) according to Dutch law, article 10a of the “Wet op de Dierproeven” and BPRC’s Animal Welfare Body (IvD). The study was conducted under the number CCD 028K. Approval by the Dutch Committee on the use of Genetically Modified Organisms to use MVA-S was granted (GGO IG 19-257_III-007). At first vaccination, the age of the animals ranged from 5 to 12 years old, and they weighed 5.6 to 9.9 kg.

Four weeks before the study start, a telemetric device (AnipillV2 temperature implant, 1.7 g, BodyCAP, Hérouville Saint Clair, France) was surgically placed in the abdominal cavity of the animals, to accurately record/transmit body temperature every 15 min. The normal circadian pattern was recorded over a 7-day period (days 8–14) before the first immunization, and for each 15-min time period of the day the mean value and 95% confidence interval were calculated. These values (mean plus 95% confidence interval) were subtracted from the temperatures recorded after immunization or infection, as described previously (32, 33). Body weight was measured every time the animals were sedated for biotechnical procedures. General behavior and stool consistency were checked daily during the immunization period. During the course of SARS-CoV-2 infection, animals were checked twice a day and scored for clinical symptoms according to a previously published scoring system (skin and fur abnormalities, posture, eye and nasal discharge, sneezing and coughing, and respiration rate) (34). A numeric score of 35 or more was predetermined to serve as an endpoint and justification for euthanasia.

### MVA-S Vaccine

The MVA-S vaccine candidate expresses a human codon-optimized full-length SARS-CoV-2 S protein (Wuhan strain), and its generation was previously described (16). The MVA-S vaccine candidate was manufactured according to current Good Manufacturing Practice by the company Biofabri (Spain), and its generation is a cost-effective system that could be scaled up for larger production. The MVA-S virus was grown in cultured chicken cells (DF-1), harvested, clarified and purified by Tangential Flow Filtration, vialed, and stored at -15°C–30°C. The MVA-WT virus was grown in DF-1 cells and purified by centrifugation through two 36% (wt/vol) sucrose cushions in 10 mM Tris–HCl (pH 9). MVA virus titers were determined by immunostaining, as previously described (35).

### Study Schedule, Vaccinations, and SARS-CoV-2 Challenge

Twelve animals (six per group) were immunized twice, with a 4-week interval, with 2 × 10^8^ PFUs/animal of MVA-S vaccine candidate or MVA-WT, by intramuscular route (500 µl in the deltoid muscle of both upper arms). Local reactions at the immunization sites were recorded on days 1, 2, 3, and 4 following each immunization by Draize scoring. Clinical chemistry and hematology measurements were performed to monitor systemic adverse effects. Then, 4 weeks after the second immunization, all 12 animals were exposed to 10^6^ TCID_50_ of SARS-CoV-2 (isolate BetaCoV/German/BavPat1/2020, similar to the Wuhan strain but containing the D614G mutation, European Virus Archive, Germany) *via* the combined intranasal (0.25 ml/nostril) and intratracheal (4.5 ml) route. Infection was monitored for 2 weeks, daily for the first 7 days, and then at days 10, 12, and 14 post-challenge.

### Enzyme-Linked Immunosorbent Assay

Individual serum samples obtained from rhesus macaques at weeks 0 and 4 after the first immunization, 2 weeks after the second immunization (week 6), and on days 10 and 14 after SARS-CoV-2 challenge (week 10) were tested for the presence of binding IgG antibodies against SARS-CoV-2 S and RBD proteins using an enzyme-linked immunosorbent assay (ELISA), as previously described (16). The S and RBD proteins used to coat the plates derived from the Wuhan strain (GenBank accession number MN908947.3) and were previously described (16). In the S protein (residues 1 to 1,208), the furin-recognition motif (RRAR) was replaced by the GSAS sequence, and it also contained the A942P, K986P, and V987P substitutions in the S2 portion. The RBD protein spans residues 332 to 534 of the S protein. Total binding IgG titers were measured as the last serum dilution that gives an absorbance value at 450 nm at least three times higher the absorbance of serum from week 0 (pre-immune serum).

### SARS-CoV-2 Neutralization

Live-virus SARS-CoV-2 neutralizing antibodies were measured using a microneutralization test (MNT) assay in a BSL-3 laboratory. Serially two-fold diluted serum samples in DMEM-2% fetal bovine serum (FBS) medium were incubated at a 1:1 ratio with 100 TCID_50_ of SARS-CoV-2 MAD6 isolate (having the D614G mutation in the S protein) in 96-well tissue culture plates for 1 h at 37°C. Then, mixtures of serum samples and SARS-CoV-2 were added in duplicate to Vero-E6 cell monolayers seeded in 96-well plates at 30,000 cells/well, and plates were incubated at 37°C, in a 5% CO_2_ incubator for 3 days. Then, cells were fixed with 10% formaldehyde for 1 h and stained with crystal violet. When plates were dried, crystal violet was diluted in H_2_O-10% SDS and optical density was measured in a luminometer at 570 nm. Neutralizing titer 50 (NT_50_) was calculated as the reciprocal dilution resulting in 50% inhibition of cell death following a methodology previously described (36). A WHO International Standard containing pooled plasma obtained from eleven individuals recovered from SARS-CoV-2 infection (NIBSC code: 20/136) was used for the calibration and harmonization of the serological assay detecting anti-SARS-CoV-2 neutralizing antibodies.

### Neutralization of SARS-CoV-2 Variants of Concern

The capacity of serum samples obtained to neutralize different SARS-CoV-2 VOC was tested by using SARS-CoV-2-pseudotyped vesicular stomatitis viruses (VSV) expressing SARS-CoV-2 S protein, which were produced as described elsewhere (37). The SARS-CoV-2 S variants used were S_614D, S_614G, alpha (B.1.1.7), beta (B.1.351), gamma (P.1), and delta (B.1.617.2). SARS-CoV-2 S mutant D614G was generated by site-directed mutagenesis (Q5 Site-Directed Mutagenesis Kit; New England Biolabs) following the manufacturer’s instructions and using as an input DNA a pcDNA3.1 expression vector encoding SARS-CoV-2 S_614D (16). SARS-CoV-2 VOC alpha (B.1.1.7; GISAID: EPI_ISL_608430), beta (B.1.351; GISAID: EPI_ISL_712096), gamma (P.1; GISAID: EPI_ISL_833140), and delta (B.1.617.2; GISAID: EPI_ISL_1970335) were optimized, synthesized, and cloned into pcDNA3.1 by GeneArt (Thermo Fisher Scientific, GeneArt GmbH, Regensburg, Germany). The neutralization activity of serum samples was tested by triplicates at several two-fold dilutions. For neutralization experiments, virus-containing transfection supernatants were normalized for infectivity to a multiplicity of infection of 0.5–1 PFU/cell and incubated with the dilutions of serum samples at 37°C for 1 h in 96-well plates. After the incubation time, 2 × 10^4^ Vero-E6 cells were seeded onto the virus–serum mixture and incubated at 37°C for 24 h. Cells were then lysed and assayed for luciferase expression; NT_50_ titers of neutralizing antibodies were determined as the highest serum dilution which resulted in a 50% reduction of luciferase units compared with pseudotyped viruses not incubated with serum.

Moreover, neutralizing antibodies against several live SARS-CoV-2 VOC were also measured by plaque reduction neutralization tests (PRNT), as described previously (38). SARS-CoV-2 viruses used were D614G (clade B; isolate Bavpat-1; European Virus Archive Global #026 V-03883), beta (B.1.351, GenBank accession number OM286905), and delta (B.1.617.2, GenBank accession number OM287123). In brief, serially diluted serum samples were incubated with 400 PFUs of different SARS-CoV-2 VOC. After 1 h at 37°C, the virus–serum mixtures were transferred onto the human airway cell line Calu-3 and incubated for 8 h. Subsequently, cells were fixed and permeabilized. Plaques were stained with polyclonal rabbit anti–SARS-CoV-2 nucleocapsid antibody (Sino Biological) and a secondary peroxidase-labeled goat anti-rabbit IgG (Dako). Plaques were visualized with the precipitate-forming 3,3′,5,5′-tetramethylbenzidine substrate (TrueBlue; Kirkegaard & Perry Laboratories) and enumerated on the ImmunoSpot Image Analyzer (CTL Europe GmbH). Next, 50% plaque reduction neutralization (PRNT_50_) titers were calculated.

### Protein Microarray

Recombinant proteins were produced for the S1 subunit (OC43, 229E, HKU1, NL63, MERS, SARS, and SARS-CoV-2) and S ectodomain (same as S1 except 229E) in HEK293 cells, as described before (39). A protein microarray was used to detect in serum samples binding IgG antibodies to those recombinant proteins as described before (40). Briefly, recombinant proteins were printed on nitrocellulose-coated glass slides (Sartorius, Göttingen, Germany) using a non-contact printer (Scienion, Berlin, Germany). After drying and blocking slides against non-specific binding using Blocker Blotto (Thermo Fisher Scientific, USA), serum was incubated in dilution series, starting from a 1 in 20 dilution in Blocker Blotto containing 0.1% Surfact-Amps (Thermo Fisher Scientific, USA), at 37°C for 1 h. After washing, slides were incubated for 1 h at 37°C using goat anti-human IgG (Fab specific, conjugated to AF647, Jackson ImmunoResearch, Ely, UK) 1 in 1,300 diluted in Blocker Blotto containing 0.1% Surfact-Amps. After washing, slides were dried and scanned using a PowerScanner (Tecan Group Ltd., Männedorf, Switzerland). Fluorescent values were analyzed using ImaGene 8.0 software (BioDiscovery, El Segundo, CA, USA). Titers were calculated from the inflection point of the serum dilution series at 50% of the maximum fluorescent signal using GraphPad prism (version 9.0.0, USA).

### ELISpot

Detection of specific IFN-γ- and IL-4-secreting cells was performed by ELISpot assay (U-CyTech, Utrecht, Netherlands), as described previously (41). In brief, 2.4 × 10^6^ PBMCs were stimulated for 16 h with 1 µg/ml of SARS-CoV-2 peptides, divided over two pools, covering the S glycoprotein (PepMix SARS-CoV-2 Spike glycoprotein, pp1 158 peptides, pp2 157 peptides, Cat no PM-WCPV-S-2, JPT innovative peptide solutions, Berlin, Germany). After stimulation, non-adherent cells were collected and plated out (2 × 10^5^ cells/well) in triplicate in PVDF ELISpot plates (Millipore, MA, USA) (including stimulus) that previously had been coated with either anti-IFN-γ or anti-IL-4 monoclonal antibodies (U-CyTech). The negative control was medium alone, and the positive control a PMA/ionomycin mixture. SARS-CoV-2 S-specific responses were calculated by subtracting the mean and two times the standard deviation of the number of spots measured in the medium control wells from the mean number of spots measured in the peptide stimulated wells and are expressed as spot-forming units (SFU) per million PBMCs. Spots were counted using the Automated ELISA-Spot assay video analysis systems (AELVIS GmbH, Hannover, Germany) reader with the aid of ELISpot reader system software.

### Quantification of Subgenomic mRNA

Tracheal, nasal, and anal swabs as well as BAL samples were analyzed for the presence of sgmRNA of the SARS-CoV-2 E gene using a quantitative real-time PCR, as previously described (42). Viral RNA was isolated using a QIAamp Viral RNA Mini Kit (Qiagen Benelux BV, Venlo, Netherlands) following the manufacturer’s instructions. The RT-qPCR assay was carried out using the Brilliant II QRT-PCR Core Reagent Kit, 1-Step Kit (Agilent Technologies BV, Amstelveen, Netherlands), according to the instructions provided by the manufacturer in a 25-ml volume with final concentrations of 600 nM for both primers, 200 nM for the probe, and 5 nM MgCl_2_, using 10 ml RNA, extracted from a 140-ml sample volume. RNA was reverse transcribed for 30 minutes at 50°C. Then, after a 10-min incubation at 95°C, the cDNA was amplified for 45 cycles, consisting of 30-s denaturation at 95°C, followed by a 1-min annealing-extension step at 60°C. All the reactions were carried out with an iQ5 Multicolor Real-Time PCR Detection System (Bio-Rad Laboratories BV, Veenendaal, Netherlands). The amount of RNA was quantified based on a standard curve made *via in vitro* transcription of a synthetic target sequence.

### CT-scan

Non-invasive, free-breathing, CT data were acquired pre-infection and 2, 7, and 15–16 days post-challenge using a MultiScan Large Field of View Extreme Resolution Research Imager (LFER) 150 PET-CT (Mediso Medical Imaging Systems Ltd., Budapest, Hungary). The macaques were positioned head first supine with the arms up and fixated in a vacuum pillow. A single CT of the chest takes 35 s by which respiratory motion is inevitable. To mitigate the impact of respiratory motion and improve the image quality, respiratory gating was applied. The respiratory amplitude was detected with a gating pad placed next to the umbilicus. For the final reconstruction, the inspiration phases were automatically selected using MATLAB (43).

CT scans of the lung were evaluated blindly by two experienced CT operators and scored, as described previously (44, 45). Quantification of CT lesions was performed based on the sum of the lobar scores. The score was normalized for preexisting abnormalities scored on day 0. The degree of involvement in each zone was scored as 0 for no involvement, 1 for <5%, 2 for 5%–24%, 3 for 25%–49%, 4 for 50%–74%, and 5 for ≥75% involvement. An additional increase or decrease of 0.5 was used to indicate alterations in CT density of the lesions. By using this scoring system, a maximum score of 35 for could be reached for the combined lobes per timepoint.

### Lung Histopathology

Tissue samples from all pulmonary lobes, the upper respiratory tract (nasal mucosa, oro/nasopharynx, trachea, left and right bronchus), heart, kidney, liver, ileum, and colon were collected for histopathology and preserved by immersion in 10% neutral-buffered formalin for 72 to 96 h. Specimens for microscopic examination were processed and embedded in paraffin, and sections of 4 µm were stained with hematoxylin and eosin (H&E). The lesions were quantified as follows: 0 = no lesions, 1 = minimal, 2 = mild, 3 = moderate, 4 = severe, using a pulmonary histopathology scoring system for SARS-CoV-2 infection in macaques, as described (46).

### Cytokines and Chemokines

Serum samples and BAL fluid were tested for the presence of cytokines and chemokines CXCL9, CXCL10, CXCL11, CCL2, CCL3, CCL4, CCL5, CCL11, IL1-β, IL-6, IL-8, IFNγ, and TNFα using the LEGENDplex assay (BioLegend LEGENDplex™ NHP Chemokine/Cytokine Panel, 13-plex, art. nr. 740317) according to the manufacturer’s manual. Samples were measured on an Aurora flow cytometer (Cytek, Fremont, CA, USA) and analyzed by using company software.

### Statistical Analyses

Statistical significance of differences between groups was calculated by using the Mann–Whitney test. A two-sided α level of 0.05 was used to determine significance. The correlation between binding IgG antibodies against RBD and virus-neutralizing antibodies and between virus-neutralizing antibodies and virus load in BAL samples was calculated by the Pearson correlation test performed on log-transformed data.

## Results

### MVA-S Vaccine Candidate Is Safe and Well Tolerated in Rhesus Macaques

**Figure 1:**
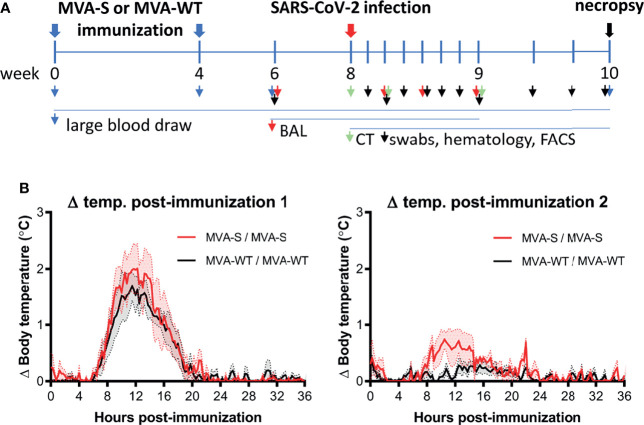
Immunization schedule in rhesus macaques. **(A)** Rhesus macaques (n = 6 per group) were immunized at weeks 0 and 4 by the intramuscular (i.m.) route with two doses of 2 × 10^8^ PFUs of MVA-S vaccine or control MVA-WT virus, as indicated (blue arrow). At week 8, all animals were challenged intranasally plus intratracheally with 1 × 10^6^ TCID_50_ of SARS-CoV-2 (red arrow). At different timepoints before and after challenge, diverse types of samples were taken, as indicated by arrowheads. All animals were sacrificed at days 15 or 16 post-challenge (black arrow). **(B)** Body temperature increase after the first (left) and second (right graph) immunization. Averages and standard error of the mean (SEM) (shaded area) are presented per group. Body temperature was recorded every 15 min. Normal 24-h body temperature before immunization (mean of 8–14 days before immunization) was subtracted from post immunization body temperature of each individual animal.

To test the safety, immunogenicity, and protective capacity of the MVA-S vaccine candidate expressing the SARS-CoV-2 full-length S protein, we immunized rhesus macaques (n = 6/group) with the MVA-S vaccine at weeks 0 and 4 using an intramuscular dose of 2 × 10^8^ plaque-forming units (PFUs)/animal. Macaques inoculated with MVA-WT were used as a control group (**Figure 1A**). At week 8 (4 weeks after the boost), all animals were challenged intranasally and intratracheally with live SARS-CoV-2 (BetaCoV/German/BavPat1/2020 isolate; 1 × 10^6^ TCID_50_/animal).

To initially determine the safety of the vaccine administration, different parameters were evaluated during the immunization period. The results showed that the MVA-S vaccine candidate was well tolerated with no adverse events detected. Local reactions at the immunization site were not observed during the observation period of 4 days after each immunization. Animals showed normal behavior and appetite, and the body weight remained stable during the immunization period. Overall, hematology and clinical chemistry levels were in the normal range (data not shown). Although body temperature increased by 1°C–2°C 12 h after the first immunization, this increase was similar in both MVA-S- and MVA-WT-inoculated animals and only transient, as normal body temperatures were reached after approximately 20 h (**Figure 1B**, left panel). After the second immunization, the temperature increase showed similar kinetics in both MVA-S and MVA-WT groups as after the first immunization, but at a much lower magnitude (**Figure 1B**, right panel).

### MVA-S Vaccination in Rhesus Macaques Induces Robust Binding IgG Antibodies Against S and RBD, Potent Titers of Neutralizing Antibodies, and T-Cell Immune Responses

**Figure 2:**
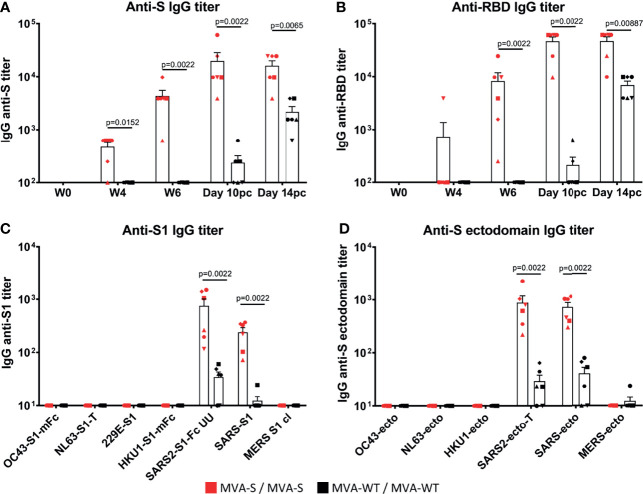
MVA-S elicited SARS-CoV-2-specific binding IgG antibodies. ELISA IgG antibody titers to SARS-CoV-2 S protein **(A),** and RBD **(B)** in time, and IgG antibody titers at 14 days post-challenge as measured by array analysis to S1 domain **(C)** and ectodomain **(D)** of the S protein from several coronaviruses (common cold α [229E, NL63] or β [OC43, HKU1] coronaviruses; SARS-CoV-2, SARS-CoV-1, and MERS) of MVA-S (red) and MVA-WT (black) immunized macaques. Each animal is represented by a symbol. Mean and SEM are shown in columns for each group of animals. Significant differences between the groups are indicated in the graph by a horizontal line and p-value (Mann–Whitney test).

Next, we analyzed the SARS-CoV-2-specific immunogenicity elicited in rhesus macaques by the MVA-S vaccine candidate at weeks 0, 4, and 6 (post-vaccination) and at 10 and 14 days post-challenge (pc). Four weeks after the first MVA-S immunization, five out of six MVA-S-vaccinated animals showed IgG antibodies to SARS-CoV-2 S protein, which were significantly boosted to high levels by the second MVA-S immunization in all macaques (week 6; 2 weeks after the second dose) (**Figure 2A**). IgG antibodies to the RBD were mainly induced after two MVA-S immunizations, but with high levels and in all animals (**Figure 2B**). MVA-WT immunization did not induce SARS-CoV-2 binding IgG antibodies. After SARS-CoV-2 challenge, binding IgGs to S and RBD proteins increased (day 10 pc) in MVA-S-immunized macaques but then did not increase further (day 14 pc) (**Figures 2A, B**). In MVA-WT animals, these SARS-CoV-2-binding IgGs were of a significantly lower magnitude (**Figures 2A, B**). This increase in SARS-CoV-2-specific humoral responses after challenge reflected an anamnestic response, due to a SARS-CoV-2 breakthrough infection. Further profiling of SARS-CoV-2-specific binding antibody responses measured at day 14 pc by protein microarray showed that responses were directed against the S1 domain, as well as against the ectodomain of the S protein, in agreement with the ELISA data (**Figures 2A, B**), and were of a significantly higher magnitude in MVA-S-immunized macaques than in animals that received MVA-WT (**Figures 2C, D**). Furthermore, positive cross-reactive antibody responses were detected against the SARS-CoV S1 region and ectodomain of the S protein, while almost no reactivity against Middle East respiratory syndrome coronavirus (MERS-CoV) and no reactivity against common cold α (229E, NL63) or β (OC43, HKU1) coronaviruses was observed (**Figures 2C, D**).

**Figure 3:**
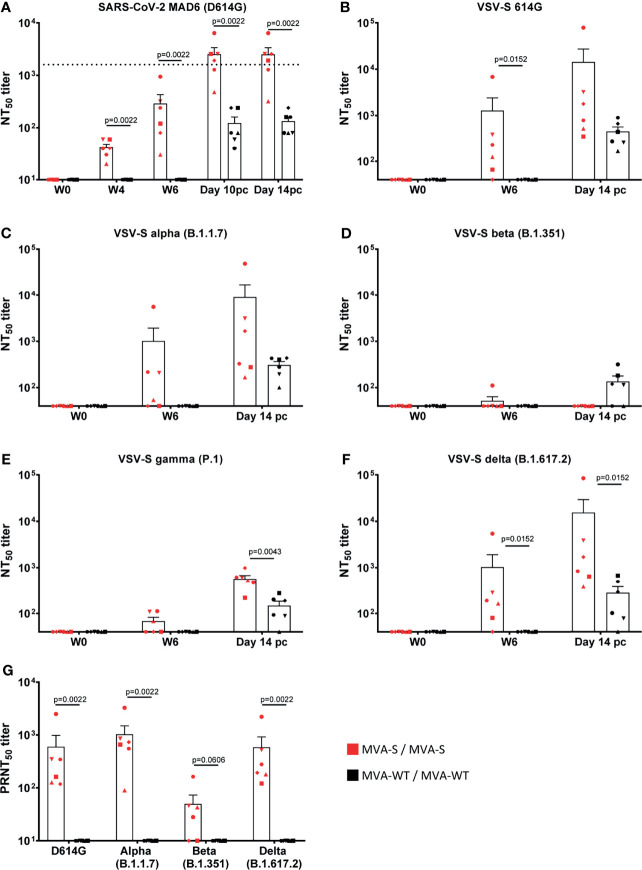
MVA-S vaccination induced SARS-CoV-2 neutralizing antibodies. SARS-CoV-2 neutralizing NT_50_ antibody titers to SARS-CoV-2 strain MAD6 (having the D614G mutation) **(A)**, VSV-luciferase recombinant viruses pseudotyped with SARS-CoV-2 Spike_614G **(B)**, alpha **(C)**, beta **(D)**, gamma **(E),** and delta **(F)** VOC at different timepoints, and live SARS-CoV-2 D614G and VOC alpha, beta, and delta **(G)**. Horizontal dotted line indicates level of neutralizing antibody titers measured in a human convalescent sera standard (NIBSC 20/136) **(A)**. Each animal is represented by a symbol. Mean and SEM are shown in columns for each group of animals. Significant differences between the groups are indicated in the graph by a horizontal line and p-value (Mann–Whitney test).

Neutralizing antibodies against live SARS-CoV-2 MAD6 strain (having the D614G mutation) were already detected in all animals after one MVA-S immunization (week 4) and again were highly boosted by the second MVA-S immunization (**Figure 3A**). Similar results were obtained against Wuhan strain (data not shown). At week 6, binding and neutralizing antibody titers showed a positive correlation in MVA-S-immunized animals (r = 0.97, p = 0.002, Pearson correlation test, log-transformed data). MVA-WT immunization did not induce SARS-CoV-2-neutralizing antibodies. After SARS-CoV-2 challenge, there was an enhancement in neutralizing antibody titers in MVA-S-immunized animals (**Figure 3A**). While after infection neutralizing antibodies were also observed in the non-vaccinated animals, they were significantly lower than in the MVA-S-vaccinated animals (10 pc and 14 pc) (**Figure 3A**). Neutralizing activity against VOCs was next assayed initially by using VSV-luciferase recombinant viruses pseudotyped with SARS-CoV-2 S_614D (parental Wuhan strain), S_614G, alpha, beta, gamma, and delta S variants. Animals immunized with MVA-S showed at week 6 comparable neutralizing antibody titers against the Wuhan S_614D (data not shown), Wuhan S_614G, and the alpha and delta variants, while lower titers against the gamma variant and no apparent neutralization of the beta variant were observed (**Figures 3B–F**). Neutralization assays using live VOC confirmed these results and showed that at week 6 post-boost vaccination with MVA-S, similar higher levels of neutralizing antibodies were elicited against SARS-CoV-2 D614G and VOC alpha and delta, but lower levels of neutralizing antibodies that were now detected against VOC beta (**Figure 3G**). Neutralizing antibodies against all VOC, except for the beta variant, increased after SARS-CoV-2 challenge (at day 14 pc) and were higher in MVA-S-immunized animals than in MVA-WT animals (**Figures 3B–F**).

**Figure 4:**
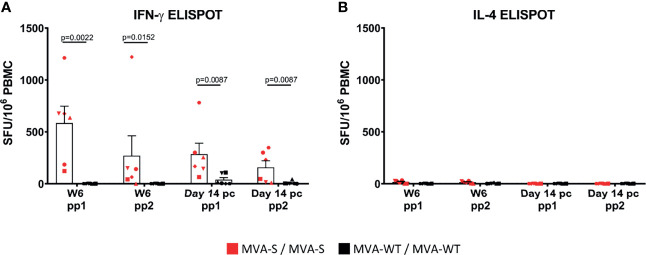
SARS-CoV-2-specific cell immune responses are induced by MVA-S. S-specific IFN-γ **(A)** and IL-4 **(B)** cell responses (directed against pp1 and pp2 peptide pools) 2 weeks post second immunization (Week 6) and 2 weeks post-challenge (day 14 pc) of MVA-S (red) and MVA-WT (black) immunized individual macaques, as measured by ELISpot assay. Each animal is represented by a symbol. Mean and SEM are shown in columns for each group of animals. Significant differences between the groups are indicated in the graph by a horizontal line and p-value (Mann–Whitney test).

Next, the analysis of SARS-CoV-2-specific T-cell immune responses elicited by MVA-S after stimulation of PBMCs with S peptide pools in an ELISpot assay showed that at 2 weeks after the second immunization (week 6), MVA-S, in contrast to MVA-WT, induced high levels of SARS-CoV-2-specific IFNγ-secreting cells that were mainly directed to the first part of the S protein (peptide pool 1, pp1) (**Figure 4A**). Remarkably, no induction of SARS-CoV-2-specific IL-4-secreting cells was observed (**Figure 4B**), reflecting a Th1-dominant response, and not a Th2 response. At 2 weeks post-challenge (day 14 pc), SARS-CoV-2-specific IFNγ-secreting cells were slightly, but not significantly, decreased in MVA-S-vaccinated macaques compared to the pre-challenge levels (**Figure 4A**). MVA-WT animals developed only very low SARS-CoV-2-specific IFNγ responses after challenge (**Figure 4A**). Again, after challenge no SARS-CoV-2-specific IL-4-secreting cells were produced (**Figure 4B**).

### MVA-S Protects From SARS-CoV-2 Infection and Replication in Lungs and Throat of Vaccinated Rhesus Macaques

**Figure 5:**
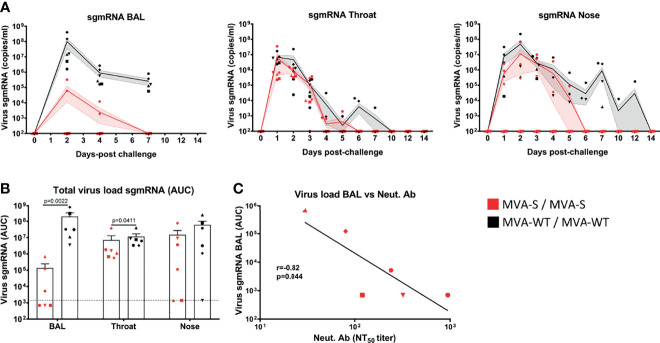
Control of SARS-CoV-2 replication by MVA-S vaccine candidate. **(A)** SARS-CoV-2 sgmRNA load (copies/ml) in lung (BAL), throat, and nose of MVA-S (red) and MVA-WT (black) immunized macaques (symbols) at different days post-challenge. Averages and SEM per group are indicated by lines with shaded areas. **(B)** Total SARS-CoV-2 sgmRNA load in BAL, throat and nose of MVA-S (red) and MVA-WT (black) immunized animals over time, as measured by the area under the curve (AUC). Individual animals (symbols), averages, and SEM are shown in columns and bars for each group of animals. Significant differences between the groups are indicated in the graph by a horizontal line and p-value (Mann–Whitney test). **(C)** Correlation between total sgmRNA SARS-CoV-2 load in BAL and neutralizing antibody titers measured at week 6 against the SARS-CoV-2 MAD6 strain (having the D614G mutation in the S protein) in animals immunized with MVA-S. The correlation was calculated by Pearson correlation test on log-transformed data. The black line represents interpolated data, as a linear curve.

Upon intranasal and intratracheal SARS-CoV-2 challenge, we measured at different days post-challenge the levels of viral subgenomic mRNA (sgmRNA) to determine the presence of replicating virus in the lung (BAL fluid), throat, and nose swabs (**Figure 5**). All MVA-WT control animals became SARS-CoV-2 infected, as SARS-CoV-2 sgmRNA was detected at high levels in BAL and throat swabs of all animals, and in nose swabs from five out of six animals, with a peak of sgmRNA at day 2 pc that then declined with time (**Figure 5A**). In contrast, all MVA-S-immunized macaques controlled SARS-CoV-2 replication. In BAL, three out of six of the MVA-S-immunized animals remained completely SARS-CoV-2 negative during the 7 days postinfection follow-up, while the other three macaques already had reduced virus levels in the lung at days 2 and 4 and cleared at day 7 pc. Instead, five out of six MVA-WT animals still showed a high virus load in BAL at day 7 pc (**Figure 5A**, left). In the throat, SARS-CoV-2 was cleared more rapidly in MVA-S-immunized animals (day 6 pc) than in the MVA-WT animals (day 10 pc) (**Figure 5A**, middle). Similarly, in nose swabs, the virus was cleared more rapidly in MVA-S-immunized animals than in MVA-WT animals. Two out of six of the MVA-S-immunized animals had no detectable virus at any time point after challenge, and the other four animals became virus negative at day 6 pc. Instead, at day 7 pc four of the six MVA-WT animals were still virus positive (**Figure 5A**, right). One of the six MVA-WT animals remained virus positive in nose swabs until day 12 pc. Moreover, MVA-S immunization significantly reduced the total SARS-CoV-2 sgmRNA loads in BAL and throat, as measured by the area under the curve (AUC) in time (**Figure 5B**). The SARS-CoV-2 sgmRNA load in BAL was negatively correlated with MVA-S vaccine-induced neutralizing antibody titers (at week 6) (r = 0.82, p = 0.044, Pearson correlation test, log-transformed data) (**Figure 5C**).

Moreover, at the time of euthanasia (14 days post-challenge), several body organs and tissues were harvested (nasal mucosa, oro/nasopharynx, trachea, left and right bronchus, seven lung lobes, heart, kidney, liver, gastrointestinal tract (ileum and colon) and cerebrospinal fluid) and processed for SARS-CoV-2 viral load analysis by RT-qPCR measuring the sgmRNA (**Supplementary Table 1**). At that time, most of the tissues were virus negative. In only 2 animals (one from each group), a very small amount (10^3^ copies/gram tissue) of SARS-CoV-2 sgmRNA was detected in one of the seven lung lobes. All other tissues from these and other animals were virus negative (**Supplementary Table 1**). Fecal samples were not examined for the presence of SARS-CoV-2 sgmRNA during the experiment, since the animals were housed in pairs, and it would be difficult to distinguish between stools. Moreover, anal swabs were taken before challenge and at 1, 2, 3, 4, 5, 6, 7, 10, 12, and 14 days post-challenge, and replicating virus was not found in anal swabs at any time point, as measured by SARS-CoV-2 sgmRNA analysis (**Supplementary Table 2**). However, at days 2 and 3 post-challenge, low levels (10^2^–10^4^ copies/ml) of genomic SARS-CoV-2 RNA were detected in anal swabs from three MVA-WT control animals and at day 3 in two MVA-S-vaccinated animals (10^2^ copies/ml) (**Supplementary Table 2**), most probably representing virus shedding.

### MVA-S Immunization Protects Against SARS-CoV-2-Induced Temperature Increase and Lung Pathology

**Figure 6:**
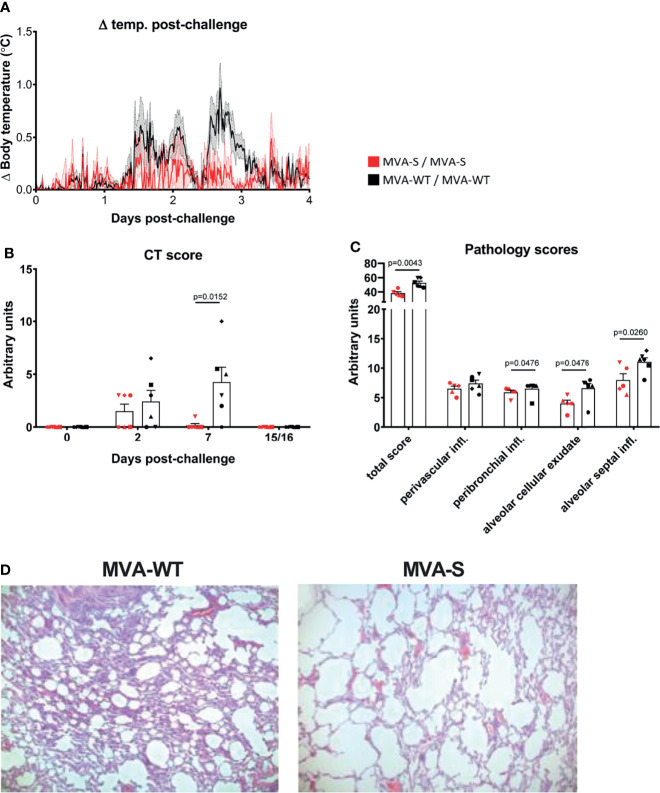
Immunization with MVA-S reduced lung pathology. **(A)** Body temperature increase after SARS-CoV-2 challenge. Averages and SEM (shaded area) are represented per group. Body temperature was recorded every 15 min. Normal 24-h body temperature before challenge (mean of 7 days) was subtracted from post-challenge body temperature of each individual animal. Body temperature changes caused by sedation procedures are not included. **(B)** CT scores of MVA-S vaccinated (red) and MVA-WT control (black) animals at days 0, 2, 7, and 15/16 post SARS-CoV-2 challenge. Individual animals (symbols), averages, and SEM are shown in columns and bars for each group of animals. Maximum score per timepoint is 35. **(C)** Lung pathology score. Shown is the total pathology score as well as the scores for perivascular inflammatory infiltrates, peribronchiolar inflammatory infiltrates, alveolar cellular exudate, and alveolar septal inflammatory cells of MVA-S-vaccinated (red) and MVA-WT control (black) animals. Scores for animal r10067 (vaccinated with MVA-S) were excluded because this animal showed non-specific vascular congestion, caused by the pentobarbital used for euthanasia. The maximum total score is 336, and the maximum score per individual parameter is 28. Significant differences between the groups are indicated in the graph by a horizontal line and p-value (Mann–Whitney test). **(D)** Lung histopathology. Lung histopathology on day 15 after infection. Shown is H&E staining on left middle lung lobe of animal r15067 (control MVA-WT) and animal r10003 (MVA-S). Original magnification ×50.

After SARS-CoV-2 challenge, no significant clinical symptoms, changes in behavior, or weight loss were observed in any of the animals at any timepoint (**Supplementary Tables 3, 4**). However, in MVA-WT animals, a slight body temperature increase was observed at 1–3 days pc that was not observed in MVA-S-immunized animals (p = 0.0357, Mann–Whitney test) (**Figure 6A**).

Next, we measured lung pathology after challenge by computed tomography (CT). Areas with increased lung density were scored semiquantitatively, as described (44, 45, 47). Modest lesions were observed at day 2 pc in four of the MVA-WT- and three of the MVA-S-vaccinated animals. These lesions were almost entirely resolved by day 7 pc in MVA-S-vaccinated animals, while lesions were still present in five of the six MVA-WT control animals (**Figure 6B**, p = 0.0152 Mann–Whitney; **Supplementary Figure 1**). At the time of euthanasia (day 15 or 16 pc), all lung lesions had resolved in all animals.

After euthanasia, one of the MVA-S-vaccinated animals (r10067) showed marked vascular congestion with moderate alveolar edema and moderate to severe alveolar hemorrhage, which were demonstrated to be typical barbiturate-euthanasia-induced artifacts (48). Due to these artifacts, a proper assessment of COVID-19-related changes in the lungs was not possible for this animal and it was excluded from further pathological assessment. After euthanasia, at day 15 or 16 pc all 7 pulmonary lobes (upper, middle, lower, accessory right and upper, middle, lower left) were processed for histopathological analyses. The microscopic examination showed that MVA-S-vaccinated animals had lower overall lung pathology scores compared with MVA-WT control animals (**Figures 6C, D**, **Supplementary Tables 5, 6**). In particular, MVA-S-vaccinated animals showed significant decreased levels of peribronchiolar inflammatory infiltrates, alveolar cellular exudate and edema, and alveolar septal inflammatory cells, while there was no difference in perivascular inflammatory infiltrates compared to control animals (**Figures 6C, D**). In summary, these findings support a beneficial role of MVA-S vaccination in reducing lung pathology. Additionally, histopathological examination of various extrapulmonary tissues, including nasal mucosa, oro/nasopharynx, trachea, left and right bronchus, heart, kidney, liver, and gastrointestinal tract (small intestine and colon), was done. Significant histological lesions in the upper respiratory tract tissues and other examined extrapulmonary organs in control and vaccinated animals were not observed (data not shown).

### Cytokine Storm Induced After SARS-CoV-2 Challenge of Rhesus Macaques Is Controlled by MVA-S Vaccination

It has been described that in humans and NHPs, SARS-CoV-2 induced a robust expression of proinflammatory cytokines and chemokines in BAL and serum samples (20, 49–51). Thus, to know whether MVA-S vaccination could reduce the cytokine storm associated with SARS-CoV-2 infection, we have analyzed the levels of several inflammatory cytokines and chemokines in BAL and serum samples at different timepoints post-challenge.

**Figure 7:**
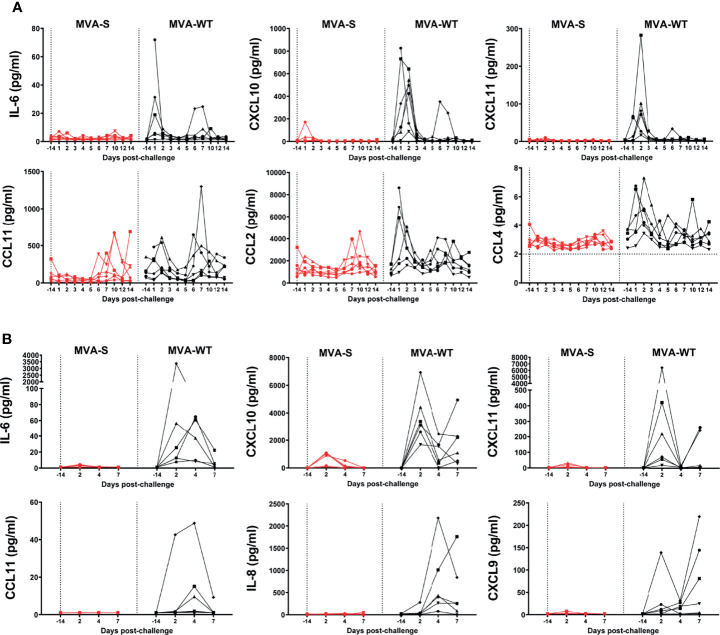
Control of SARS-CoV-2-induced cytokine storm by MVA-S vaccine candidate. **(A)** IL-6, CXCL10, CXCL11, CCL11, CCL2, and CCL4 levels in serum of MVA-S (red) and MVA-WT (black) groups in time. Horizontal dotted line indicates lower limit of detection. At day -14, the sample of one MVA-WT animal is missing. **(B)** IL-6, CXCL10, CXCL11, CCL11, IL-8, and CXCL9 levels in BAL of MVA-S (red) and MVA-WT (black) groups in time. All data are shown relative to the day of challenge (day 0, study day 56). Levels of individual values (indicated by different symbols) in time are shown. No measurements were performed on day of challenge, but data obtained at day 14 before challenge are shown.

In serum samples, there was a transient increase (mainly at days 1–2 pc) in levels of CXCL10 (IP-10), CXCL11 (I-TAC), CCL2 (MCP-1), CCL4 (MIP-1β), and CCL11 (eotaxin) in all MVA-WT control animals, while this increase was either absent or only seen at low levels in the MVA-S-vaccinated group (**Figure 7A**). IL-6 showed a peak expression on day 1 pc in three animals of the MVA-WT control group, but in none of the animals of the MVA-S vaccine group. For CCL2 and CCL11, there was a second increase from day 6 pc onward, but this was similarly observed in both groups (**Figure 7A**). IL-1β, TNFα, and IFNγ levels were below 10 pg/ml in all animals at all timepoints and did not show any clear pattern of change, while levels of IL-8 were too high and fell above the limit of detection (data not shown). CXCL9 (MIG) showed only one peak on day 10 pc in one animal of the MVA-S vaccine group and one animal of the control group (data not shown). CCL3 (MIP-1α) and CCL5 (RANTES) were observed in all pre- and post-infection sera without any specific change in expression level (data not shown).

Additionally, we also observed that MVA-S immunization protected all animals from the cytokine storm in the lung (BAL samples) (**Figure 7B**). A transient increase, peaking at day 2 pc, was observed in IL-6, CXCL10, and CXCL11 levels in animals from the control MVA-WT group, while this increase was either absent or only seen at low levels in the MVA-S-vaccinated group at any timepoint (**Figure 7B**). CCL11 levels peaked on day 4 pc in three of the six animals of the MVA-WT group, but none of the animals of the MVA-S-vaccinated group. IL-8 levels in the MVA-WT control group also peaked at day 4 pc, with no levels in the MVA-S-vaccinated group at any time point (**Figure 7B**). CXCL9 peaked on day 2 pc in one animal from the MVA-WT group, and a late increase at day 7 pc was observed in four other animals of this group, but no increase was observed in any of the MVA-S-vaccinated animals (**Figure 7B**). CCL5, IL-1β, TNFα, and IFNγ were below 10 pg/ml in all animals at all timepoints, while CCL2, CCL3, and CCL4 levels were variable and without any clear pattern of change (data not shown).

Regarding changes in leukocytes post-challenge, we observed that while the number of granulocytes transiently increased in all MVA-WT control animals, this only occurred in two of the six MVA-S-immunized animals (**Supplementary Figure 2**). The number of lymphocytes, involving T-cells, B-cells, and NK cells, showed a transient decrease in all animals of the MVA-WT- as well as the MVA-S-vaccinated group (**Supplementary Figure 2**). Monocytes and myeloid dendritic cells (DC) displayed a transient increase in CD16 and CD80 expression, respectively, in MVA-WT control animals, while the increase was smaller or absent in MVA-S-vaccinated animals (**Supplementary Figure 3**). There was a transient increase in CD69 activation marker expression on CD4 and CD8 T cells in both MVA-S-vaccinated and MVA-WT control animals, and levels of CD80 activation marker expression on NK cells were comparable between the groups (**Supplementary Figure 3**).

## Discussion

In this investigation, we evaluated the safety, immunogenicity, and efficacy in rhesus macaques of a COVID-19 vaccine candidate based on the poxvirus MVA vector expressing a human codon-optimized full-length SARS-CoV-2 S protein (MVA-S), which was previously reported to induce potent B- and T-cell immune responses and full efficacy in mice (16, 17, 19). Due to the close relatedness of NHPs to humans, inoculation of rhesus macaques with SARS-CoV-2 has been used for current vaccines as a preclinical animal model system, as it leads to a respiratory disease, with virus replication in lungs and in the upper and lower respiratory tracts (52).

The MVA-S vaccine candidate was safe and well tolerated, showing only a transient increase in temperature by 12 h post first immunization, but comparable to the increase induced by control MVA-WT animals. The transient increase in body temperature was similar to most of the current vaccines being administered worldwide, and this effect is likely related to the inflammatory response produced by the MVA vector at the site of virus inoculation, with vector expression being restricted to 24–48 h post inoculation (53, 54).

Neutralizing antibody responses are highly predictive of immune protection against symptomatic SARS-CoV-2 infection in humans (55, 56) and were described to be inversely correlated with virus production in BAL samples from macaques (57, 58). Here, we demonstrated that vaccine candidate MVA-S triggered strong humoral immune responses, mainly after two doses, as defined by binding IgG antibodies to the S and RBD components, as well as of high neutralizing antibodies against live SARS-CoV-2 expressing an S protein containing the D614G mutation (Wuhan D614G and MAD6 isolates). Neutralizing antibody titers were inversely correlated with virus production in BAL samples from MVA-S-immunized macaques. VOC alpha and delta were also well neutralized, while responses against the gamma variant were slightly lower and further lower neutralization against the beta variant was observed. This resistance could be related to the nature of mutations in the RBD domain of the beta variant. With the recent appearance of a beta variant, named Ómicron, with multiple mutations within the S protein, there is concern that the virus spreads rapidly and manifests more resistance to current vaccines. Hence, vaccines with multiple modes of action are needed. Following SARS-CoV-2 challenge, binding and neutralizing antibodies against live SARS-CoV-2 (containing the D614G mutation in the S protein) as well as against VOC alpha, gamma, and delta were further enhanced. This is most likely due to a breakthrough virus infection, reflecting an anamnestic response. Surprisingly, by the S-VSV pseudovirus assay levels of neutralizing antibodies against the beta variant were only observed after challenge in MVA-WT control animals, but not in MVA-S-immunized animals. These antibodies were apparently only triggered by virus infection and not by immunization, which is at variation with induction of broad virus-neutralizing antibody responses upon breakthrough infection in humans (59). In contrast, a recent publication describes induction of broadly SARS-CoV-2-neutralizing antibodies upon immunization of rhesus macaques with recombinant a prefusion-stabilized S protein in saponin adjuvant, while such responses were not observed in S-mRNA-vaccinated or SARS-CoV-2-infected humans or in mice immunized with S protein (60). In macaques, most of the broadly neutralizing antibodies expressed a heavy chain derived from the germline IGHV3-73 gene and targeted a relatively conserved region proximal to the RBD. This is different from the epitopes usually targeted by human antibodies. Our study could indicate that such a relatively broader response may also have been triggered by the viral infection, but this needs further investigation. Moreover, analysis of post-challenge sera showed that SARS-CoV-2-specific antibody responses were directed against the S1 region and ectodomain of the S protein, also with cross-reactive antibody responses against SARS-CoV, but almost no reactivity against MERS and no reactivity against common cold α- or β-coronavirus S proteins, as also shown in human COVID-19 (61).

The analysis of cell responses showed that S-specific IFN-γ-secreting cells predominantly directed against the S1 subunit (pp1) were induced in MVA-S-vaccinated animals 2 weeks after vaccination, but not in the MVA-WT control group. After SARS-CoV-2 challenge, cell responses were maintained in the vaccinated group with minimal responses in the control group. In contrast, almost no S-specific IL-4-secreting cells were detected, reflecting the absence of a T helper 2 response. Thus, this T helper 1 (Th1) skewed response together with the induction of virus-neutralizing antibodies implies that the potential risk for vaccine-associated enhanced respiratory disease is very low. There have been reports of adverse effects of MVA vectors expressing the SARS-CoV-1 S protein when tested in NHPs, with an antibody-dependent enhancement (ADE) effect after SARS-CoV-1 infection (62) and with liver damage in ferrets (63). However, such adverse events have not been observed in this NHP study or in any of the preclinical studies performed to date with MVA vectors expressing SARS-CoV-2 S antigens (15–22). This is likely related to the distinct biology of the SARS-CoV-2 infection and the antigenic nature of the viral protein components. The potent immune responses (both humoral and T cell) induced by MVA vectors expressing SARS-CoV-2 S antigens, as shown here and by others (15–22), will have a major impact in reducing virus loads in tissues and disease progression.

We next evaluated the extent of SARS-CoV-2 infection and replication in various tissues by measuring viral sgmRNA. In animals immunized with MVA-S, there was no detectable SARS-CoV-2 replication in BAL in three animals and virus replication was reduced in the other three animals relative to the MVA-WT control animals. Moreover, a significant reduction of viral sgmRNA was also observed in the throat of vaccinated macaques. In addition, virus was cleared faster from the throat and nasal cavity of vaccinated animals, which could be important in reducing viral spread. Hence, the high protective efficacy of the MVA-S vaccine is comparable to other vaccine modalities evaluated in macaques (57, 64, 65) or to secondary infection (66) and possibly superior to other studies where the infection may have been less stringent, as it was cleared faster in the control group (67, 68). Furthermore, a significant reduction in fever was observed in MVA-S-vaccinated animals upon challenge, as previously also reported for the Ad26.COV2-S vaccine (64).

A feature of SARS-CoV-2 infection is an enhancement of pathophysiological changes associated with disease, like pulmonary lesions with edema and induction of a cytokine storm leading to death of infected individuals (69, 70). The evaluation of lung lesions by CT at various timepoints after virus challenge showed a strong significant inhibition of lung lesions (CT scores) in all MVA-S-vaccinated animals by day 7 post-challenge, compared to MVA-WT control animals. At day 15 or 16 post-challenge, only modest histopathological changes were observed after the autopsy in all the animals of the study, where mild clinical disease was observed, which agrees with other SARS-CoV-2 infection studies in macaques (52). This is also in agreement with other studies, showing that typical pathological features of viral pneumonia in rhesus macaques (71, 72) also spontaneously resolve by day 14 postinfection (46). However, at day 15 or 16 post-challenge there was a significant reduction in interstitial septal inflammatory cells, alveolar cellular exudate, and peribronchiolar inflammatory infiltrates in MVA-S-vaccinated animals relative to control macaques. Similar mild histopathological lung lesions were described in efficacy studies in SARS-CoV-2-infected macaques previously vaccinated with SARS-CoV-2 vaccines based on mRNA (67, 73), adenovirus (68, 74), inactivated viruses (75), or MVA (20), among others.

An important feature of MVA-S vaccine efficacy was the total prevention in serum and BAL samples of the acute inflammatory cytokine response typically induced by SARS-CoV-2 infection in macaques (71, 76) that was observed in the control animals of this study. These cytokines are known to drive the development of severe COVID-19 in humans (77), and preventing such cytokine release is an important aspect of vaccine efficacy.

We and other groups have generated MVA vectors expressing the S protein of SARS-CoV-2, showing in mice or hamsters induction of humoral and T-cell immune responses and efficacy in lung tissues, throat, and nose (15–22). Moreover, the immunogenicity and efficacy were also defined in NHPs with an MVA vector expressing a prefusion-stabilized S protein, where robust humoral and cellular immune responses and high efficacy against SARS-CoV-2 infection were reported (20). Instead, our study evaluated the immunogenicity and efficacy of an MVA vector expressing a full-length non-stabilized S protein. Both the full-length non-stabilized S protein and a stabilized prefusion form of the S protein have been applied in current vaccines against COVID-19. There is currently no indication that either one of these strategies might result in lower vaccine efficacy in humans against either the Wuhan strain or the alpha or beta VOC, although in mice we have observed higher neutralization capacity against VOC of serum from animals vaccinated with a prefusion-stabilized S over non-stabilized S protein (19). Nonetheless, such differences could arise for other newly emerging VOC. We showed that MVA-S did induce neutralizing antibodies against VOC alpha, gamma, beta, and delta, while this has not yet been described for the MVA vector expressing a prefusion-stabilized S protein evaluated by Routhu et al. (20). A difference with Routhu et al. (20) was not only in the virus promoter and site of insertion of the S gene within the MVA genome, but in the dose of SARS-CoV-2 used for challenge, 5 × 10^4^ PFU/animal while we used 1 × 10^6^ TCID50/animal, which could explain the higher efficacy observed with lower doses of the challenge virus. Together, all these studies highlight the benefit of MVA vectors as vaccine candidates against SARS-CoV-2.

By the nature of the biology of the recombinant MVA vector and its impact on the host cell immune system, this type of poxvirus-based vaccine could induce, upon inoculation of preferably two doses, robust, broad, and durable protective memory B- and T-cell immune responses. In addition, it has been established that a heterologous combination of poxvirus vectors with other vaccines potently enhances the immune responses and efficacy against pathogens (26, 29–31, 78). In particular, clinical trials have shown an enhancement of immune responses against the S protein of SARS-CoV-2 by the combination of adenovirus vaccines (AstraZeneca) and mRNA vaccines (79–82); clinical studies with various combinations of vaccines are currently ongoing. As such, the MVA-S vaccine deserves a consideration to enter phase 1/2 clinical trials either alone or in combination with other vaccines.

In conclusion, our findings demonstrate that the MVA-S vaccine is safe and well tolerated in rhesus macaques, being highly immunogenic with the induction of potent levels of binding and neutralizing antibodies against several SARS-CoV-2 VOC, as well as Th1-skewed cell responses. Remarkably, this vaccine provides protection from SARS-CoV-2 challenge, reducing virus replication in the upper and lower respiratory tracts, lung pathology, and cytokine storm. All these results support the entering of the MVA-S vaccine into clinical trials to evaluate its safety, immunogenicity, and efficacy.

## Data Availability Statement

The original contributions presented in the study are included in the article/**Supplementary Material**. Further inquiries can be directed to the corresponding authors.

## Ethics Statement

The study was reviewed and approved by the Dutch “Centrale Commissie Dierproeven” (AVD5020020209404-2) according to Dutch law, article 10a of the “Wet op de Dierproeven” and BPRC’s Animal Welfare Body (IvD). The study was conducted under the number CCD 028K. Approval by the Dutch Committee on the use of Genetically Modified Organisms to use MVA-S was granted (GGO IG 19-257_III-007).

## Author Contributions

Conceptualization: PM, JG-A, GK, ME. Formal analysis: PM, JG-A, BV, MS, IK, GK, ME. Funding acquisition: JG-A, ME. Investigation: PM, JG-A, PP, AL-F, KB, DaM, ZF, GK-K, HN, RA, MS, IK, CG, EB, RS, JL, RD. Methodology: JG-A, PP, AL-F, KB, DaM, ZF, GK-K, HN, RA, LM, MS, IK, JL, RD. Resources: DoM, EP, ER, WB. Supervision: PM, JG-A, BV, EV, CG, WB, GK, ME. Validation: PM, JG-A, BV, EV, GK, ME. Visualization: PM, JG-A, GK, ME. Writing original draft: PM, JG-A, GK, ME. Writing, review and editing: all authors. All authors contributed to the article and approved the submitted version.

## Funding

This research was supported by Fondo COVID-19 grant COV20/00151 (Spanish Health Ministry, Instituto de Salud Carlos III (ISCIII)), Fondo Supera COVID-19 grant (Crue Universidades-Banco Santander), and Spanish Research Council (CSIC) grant 202120E079 (to JG-A); CSIC grant 2020E84, la Caixa Banking Foundation grant CF01-00008, Ferrovial, and MAPFRE donations (to ME); a Spanish Ministry of Science and Innovation (MCIN)/Spanish Research Agency (AEI)/10.13039/501100011033 grant (PID2020-114481RB-I00; to JG-A and ME); and internal funding from the BPRC. This research work was also funded by the European Commission-NextGenerationEU, through CSIC’s Global Health Platform (PTI Salud Global) (to JG-A and ME). RD received grants from the European Commission Horizon 2020 Framework Programme (Project VIRUSCAN FETPROACT-2016: 731868 and Project EPIC-CROWN-2: 101046084), and Fundación Caixa-Health Research HR18-00469 (Project StopEbola).

## Conflict of Interest

Author EP was employed by Biofabri.

The remaining authors declare that the research was conducted in the absence of any commercial or financial relationships that could be construed as a potential conflict of interest.

## Publisher’s Note

All claims expressed in this article are solely those of the authors and do not necessarily represent those of their affiliated organizations, or those of the publisher, the editors and the reviewers. Any product that may be evaluated in this article, or claim that may be made by its manufacturer, is not guaranteed or endorsed by the publisher.
