# Supplementary material for: Poxvirus MVA Expressing SARS-CoV-2 S Protein Induces Robust Immunity and Protects Rhesus Macaques From SARS-CoV-2
Source: Front Immunol. 2022 Mar 16;13:845887. doi: 10.3389/fimmu.2022.845887 (PMC8966779; doi:10.3389/fimmu.2022.845887)
Supplement: Supplementary file 1 [file DataSheet_1.pdf]

Supplementary information

Poxvirus MVA Expressing SARS-CoV-2 S Protein Induces Robust Immunity and Protects Rhesus Macaques From SARS-CoV-2

Petra Mooij [1,*,†], Juan García-Arriaza [2,3,*,†], Patricia Pérez [2,3], Adrian Lázaro-Frías [2,3], Babs E. Verstrepen [1], Kinga P. Böszörményi [1], Daniella Mortier [1], Zahra Fagrouch [1], Gwendoline Kiemenyi-Kayere [1], Henk Niphuis [1], Roja Fidel Acar [1], Lisette Meijer [4], Marieke A. Stammes [4], Ivanela Kondova [5], Ernst J. Verschoor [1], Corine H. GeurtsvanKessel [6], Erwin de Bruin [6], Reina S. Sikkema [6], Joanna Luczkowiak [7], Rafael Delgado [7,8], Dolores Montenegro [9], Eugenia Puentes [9], Esteban Rodríguez [9], Willy M. J. M. Bogers [1], Gerrit Koopman [1,*] & Mariano Esteban [2,*]

[1] Department of Virology, Biomedical Primate Research Centre (BPRC), Rijswijk, The Netherlands; [2] Department of Molecular and Cellular Biology, Centro Nacional de Biotecnología (CNB), Consejo Superior de Investigaciones Científicas (CSIC), Madrid, Spain; [3] Centro de Investigación Biomédica en Red de Enfermedades Infecciosas (CIBERINFEC), Madrid, Spain; [4] Department of Parasitology, Biomedical Primate Research Centre (BPRC), Rijswijk, The Netherlands; [5] Animal Science Department, Biomedical Primate Research Centre (BPRC), Rijswijk, The Netherlands; [6] Department of Viroscience, Erasmus Medical Center (MC), Rotterdam, The Netherlands; [7] Instituto de Investigación Hospital Universitario 12 de Octubre (imas12), Madrid, Spain; [8] Department of Medicine, Universidad Complutense School of Medicine, Madrid, Spain; [9] Biofabri, O Porriño, Pontevedra, Spain.

† These authors have contributed equally to this work and share first authorship
* Correspondence: mooij@bprc.nl (P.M.), jfgarcia@cnb.csic.es (J.G.-A.), koopman@bprc.nl (G.K.), mesteban@cnb.csic.es (M.E.)

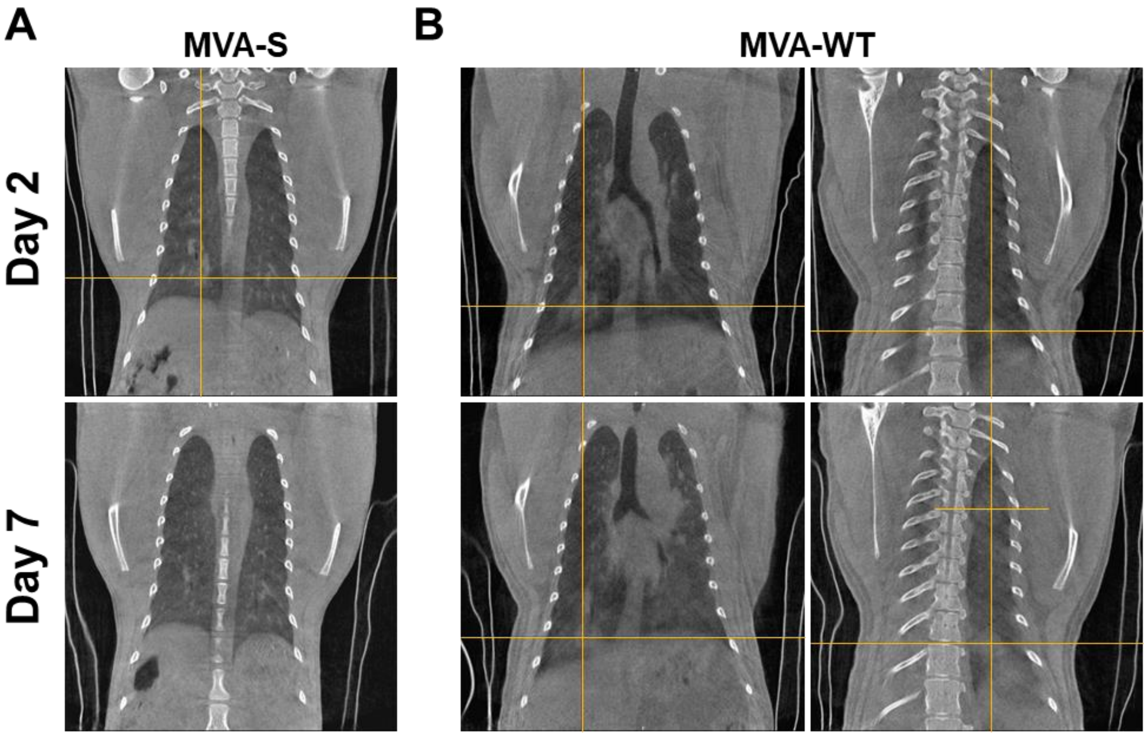

Supplementary Figure 1. Representative coronal slices of the lungs obtained at day 2 and 7 post-infection of an MVA-S vaccinated (A) and an MVA-WT control animal (B). For both days a comparative slice is used. The center of the lesions, when present is marked with a yellow colored cross-hair. **(A)** At the center of the lower right lobe of an MVA-S-vaccinated macaque a consolidation is formed at day 2 post-infection which is resolved at day 7. This in contrast to the MVA-WT-inoculated animal visualized in **B)**, where the lesion, a consolidation, found at the same location as in A, is still present at day 7. In addition, in the left lung of the same animal the consolidation in the lower lobe is increased in size and a new ground glass opacity is formed in the upper lobe.

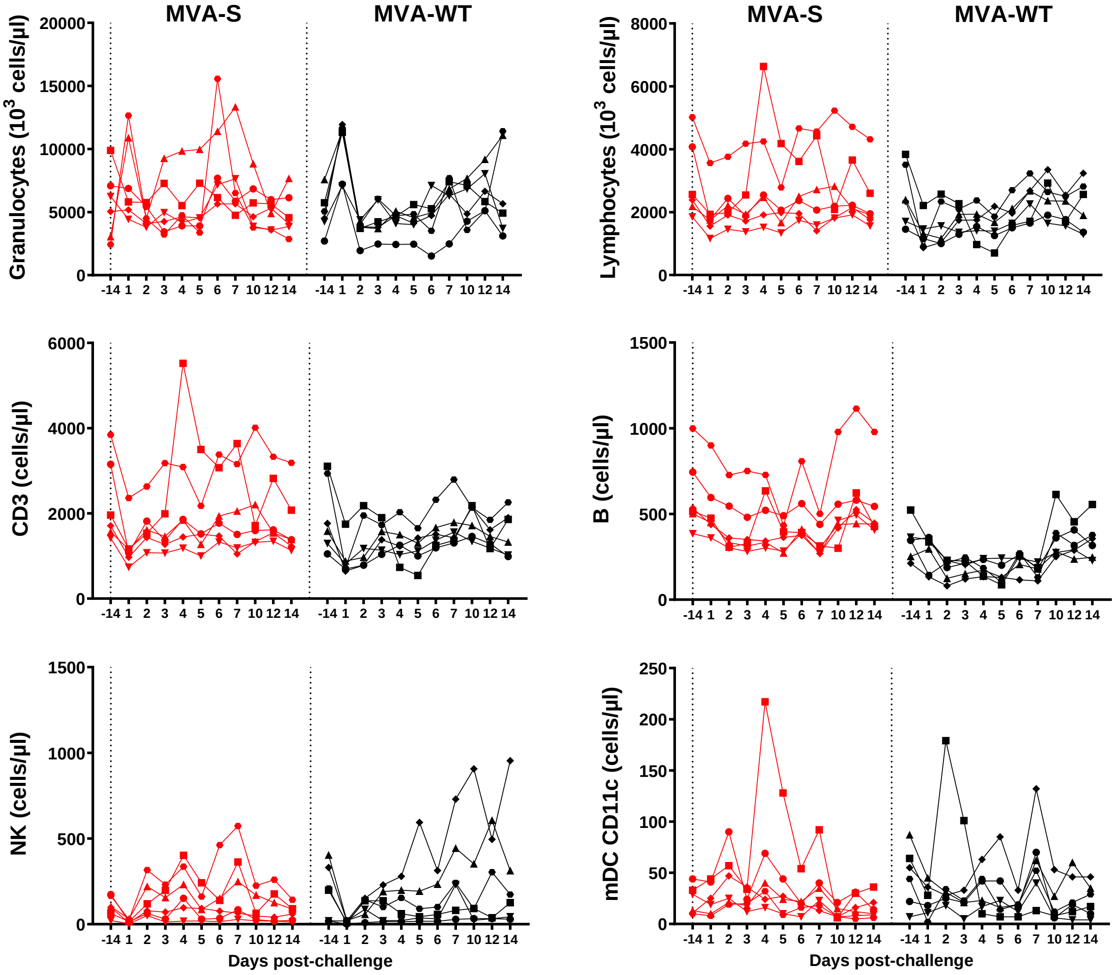

Supplementary Figure 2. Cell populations induced after SARS-CoV-2 challenge in vaccinated macaques. Granulocyte, lymphocyte, CD3 T cell, B cell, NK cell and mDC CD11c counts after virus challenge in the MVA-S vaccine group (left panel, red) and in the MVA-WT control group (right panel black) in time. Data are shown relative to the day of challenge (day 0, study day 56). No measurements were performed on day of challenge, but data obtained at day 14 before challenge are shown.

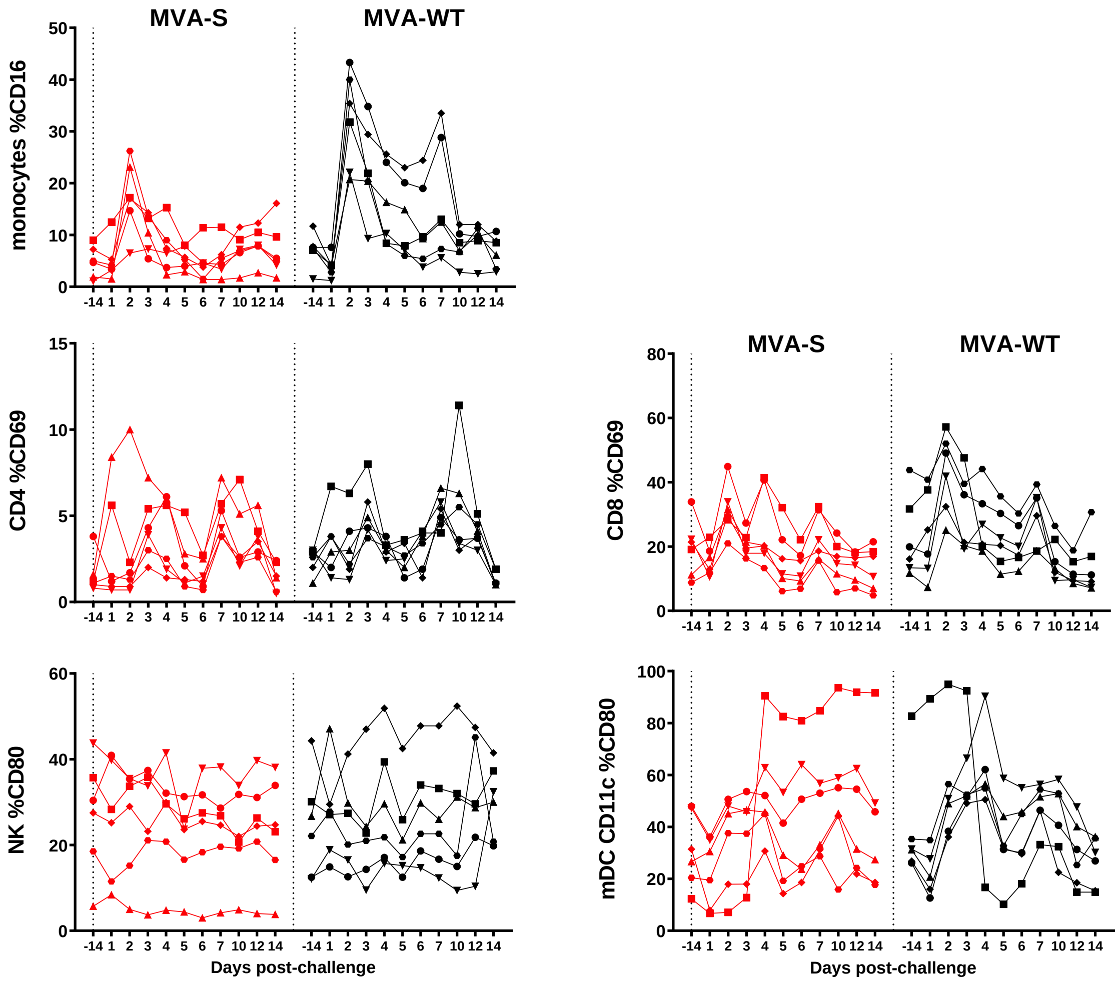

Supplementary Figure 3. Activated cell populations induced after SARS-CoV-2 challenge in vaccinated macaques Activation of monocytes (%CD16), CD4 and CD8 T-cells (%CD69), NK cells and mDC CD11c (%CD80) after virus challenge in the MVA-S vaccine group (left panel, red) and in the MVA-WT control group (right panel, black) in time. Data are shown relative to the day of challenge (day 0, study day 56). No measurements were performed on day of challenge, but data obtained at day 14 before challenge are shown.
